# Genetic Diversity and Population Structure of Selected Ethiopian Indigenous Cattle Breeds Using Microsatellite Markers

**DOI:** 10.1155/2023/1106755

**Published:** 2023-01-14

**Authors:** Shelema Kelbessa Bora, Tesfaye Sisay Tessema, Gebrerufael Girmay

**Affiliations:** ^1^Ethiopian Institute of Agricultural Research, National Agricultural Biotechnology Research Center, P.O. Box 249, Holeta, Ethiopia; ^2^Addis Ababa University, Institute of Biotechnology, P.O. Box 1176, Addis Ababa, Ethiopia

## Abstract

**Background:**

In Ethiopia, livestock contributes 45% of agricultural GDP. Despite the economic role played by the sector, there have been little efforts to genetically improve the indigenous cattle. Morphological characterization of selected Ethiopian indigenous cattle has been made for (Bonga, Jimma, and Kerayu) cattle types. But, the selected indigenous cattle were not characterized at molecular level (genetic diversity information). Hence, this work was initiated to detect and determine the genetic diversity and population structure of selected Ethiopian indigenous cattle ecotypes using microsatellite markers.

**Results:**

Different alleles were identified (131) and thirty-three of these alleles were unique to specific ecotypes. All loci used were informative with PIC values ranging from 0.5 (TGLA126) to 0.84 (ETH10) with a mean of 0.70 per locus. The Shannon information index ranged from (I = 1.02) ILST006 to (I = 1.63) ETH10 with an average of 1.28 revealing there is genetic diversity. Moreover, analysis of molecular variance (AMOVA) revealed 84% genetic variation within a population and 13% variation among populations. The value of *F*-statistics (Fst) (0.129 = 13%) indicated that there was moderate genetic differentiation among ecotypes. The (UPGMA) revealed, Bonga and Jimma clustered together while Kerayu cattle were relatively distinct, Principal coordinates analysis (PCOA) and structure analysis grouped the individual into different clusters confirming the presence of ecotype admixture due to geographical origins and uncontrolled mating.

**Conclusion:**

In general, this study has successfully characterized the genetic diversity and population structure of Bonga, Jimma, and Kerayu cattle ecotypes using high polymorphic/informative microsatellite markers. According to this study, Kerayu cattle have high AR and PA when compared to Bonga and Jimma cattle populations. So, the Kerayu population is more diverse than others and it is the hotspot for genetic diversity study. The generated information is very relevant for breeder and genetic conservation.

## 1. Introduction

In several parts of developing countries including Ethiopia, livestock production is the major and achieving good living standards. From Africa, Ethiopia is ranked first and among the top 10 countries in the world in major farm animal populations [1].

The livestock sector in our country has advantages for agricultural production, and domestic farm animals, in particular, are very significant, accounting for 30–40% of the food and agricultural sectors' economic value. In Ethiopia, livestock contributes 45 percent of agricultural GDP, 18 percent of overall GDP, and 19 percent of export earnings [2]. There are diverse numbers of indigenous cattle breeds in Ethiopia. According to the Ethiopian Biodiversity Institute [3] report, there are more than 28 native cattle breeds/ecotypes that have been recognized to exist in the country. Cattle diversity in our country is due to the prevalence of both Bos taurus and Bos indicus cattle in the country. Ethiopia is also well known for its varied climatic and topographic environments. This has contributed to several distinct cattle breeds that have evolved [4].

Despite the global economy's commitment and diversity, there is a rapid depletion of farm animal genetic capital [5]. Among the factors, cross breeding, inbreeding, breed replacement, starvation, drought, and conflict are the factors which affects genetic diversity of indigenous cattle [6].

As a result, understanding farm animal genetic diversity is needed to contribute to meeting current production needs in different settings, enable continued genetic development, or promote rapid adaptation to evolving environments (harsh environments), as well as to be used for breed selection based on their genetic contents for the dairy industry and beef industry [2].

Therefore, molecular characterization helps for effective conservation and long-term use strategies. Some phenotypic and genotypic characterization research has been done on Ethiopian indigenous cattle. However, so far identification and characterization of Ethiopian livestock resources are not exhaustive. There are, however, certain indigenous breeds that are known to exist at various levels of threat, interbreeding with other indigenous breeds and changes in production practices, for example, Sheko cattle. In addition, the cattle breeds Begait, Irob, Ogaden, Afar, and Borana are all threatened in some way [2, 3]. As a result, the identification of indigenous cattle breeds, as well as their distinct characteristics, should be prioritized. That should be a prerequisite for developing conservation and sustainable utilization programs to assist indigenous breeds to compete in the future with limited production resources such as land, feed, labor, and capital, knowing the genetic diversity of Ethiopian indigenous cattle is for improvement and conservation purposes. As an example, particularly in terms of disease-tolerant cattle population in the country (for example, Sheko), cattle have been reported as tolerant to trypanosomosis. Not all ancestral Ethiopian cattle have been analyzed at the molecular level. Some attempts have been made in the country to classify and recognize cattle genetic resources. However, those attempts are not enough to give the actual cattle genetic diversity of the country's indigenous (local) cattle breeds [3].

Characterization attempts were mainly focused on farms, phenotypic features of genetic properties, and their products such as meat and milk, among other things [3]. Some molecular characterization of selected indigenous cattle breeds has been done, and it is not sufficient to say all Ethiopian indigenous cattle have been characterized at a molecular level. So far, the genetic diversity of Ethiopian indigenous cattle such as Horro, Guraghe, Arsi and Abigar, Zebu, Boran, Ambo, Adwa, Ogaden, Zebu Sanga, Fogera, Sanga, Raya-Azebo, Danakil, and African taurine Sheko have been analyzed using RAPD, microsatellite (SSR), and single nucleotide polymorphism (SNP) [6–8]. Even though cattle ecotypes like Kerayu are claimed for resistance to heat and tolerant to drought, they are not yet characterized. Similarly, the Bonga and Jimma ecotypes have variable coat colors, which help the ecotype to adapt to the very hostile environment and heat stress, and the Jimma ecotype is resistant to disease and high milk production when compared to both ecotypes, respectively. Morphological characterization has been done for Bonga, Jimma, and Kerayu cattle. However, molecular characterization has not been done for the three selected indigenous cattle. Therefore, the main aim of this study was to characterize the genetic variability of these three Ethiopian indigenous cattle ecotypes (Bonga, Kerayu, and Jimma) cattle using microsatellite markers.

## 2. Objective

- To identify the extent of genetic diversity among three indigenous cattle breeds
- To determine the population structure of the selected indigenous cattle breeds

## 3. Materials and Methods

### 3.1. Study Area

Generally, the study was conducted in three areas that have different agroecological setups (Figure S1). The first study area was Jimma Zone, Southwestern Ethiopia. The area is situated approximately between 360 10′E longitude and 70 40′N latitude at an elevation ranging from 880 to 3360 meters above sea level. The zone has an agroecological setting of highlands (15%), midlands (67%), and lowlands (18%) [9].

The second study area was in the Fentale district located in the East Shoa zone of Oromia, the southern part of the northern Rift Valley of Ethiopia. The area falls within an altitude range of 800–1100 masl. However, there are high peaks on the Fentale mountain from which the district derives its name, reaching up to 2007 masl [10]. It is found at a distance of about 200 km east of the capital city of Ethiopia, Addis Ababa, on the way to Harar. It is affected by recurrent droughts due to disrupted rainfall patterns. The total land area of the district is 1170 km^2^. The third study area was Southern Nations Nationalities and Peoples Regional State, Kaffa administrative Zone of Chena woreda. It is situated in the southwestern part of Ethiopia (7°34′N latitude and 37°6′E Longitude) and with an altitude range of 1851–1900 mean above sea level.

### 3.2. Description of the Breeds

#### 3.2.1. Jimma-Type Cattle

Zenga type (Zebu), mainly distributed in the Jimma zone with medium horns and big body frames. Daily milk yield per day is expected to be about 1.92 liters. Among the Jimma zone, the Dedo district is the highest in milk production and lactation length (3–8) months [11]. This may be due to management and genetic makeup used for draft power, milk, and meat. Coat color is red, black, and mixed white red and resistant to heat and disease.

#### 3.2.2. Kerayu Cattle

Sanga type breed and distributed in the Kerayu area of Eastern Shoa, mainly for milk, meat, saving, and dowry, with an average body weight of 300.4 kg and 249.9 kg male and female, respectively [9]. They are well adapted to the hot environmental situation with a straight profile, long thin legs, and long horns plain, patchy, and spotty.

#### 3.2.3. Bonga Cattle

Bonga cattle are a Zebu type found in the northern, western, and northwest parts of the Kaffa zone. It originated from around Horro Guduru of Wollega and is used for draft power, milk production, and meat production. The average lactation length of the Bonga cattle ecotype is about 8.5 months and the daily milk yield per cow is about 1.98 liters [10]. The coat color of the Bonga cattle ecotype is red, black, light red, grayish, patchy, and spotted. They have downward, upward, mix, and forward horn orientations.

### 3.3. Blood Samples

A total of 72 genotype were collected from (Bonga, Jimma, and Kerayu) and 24 from each.

Genotype from the same administrative zone was considered as one population with assumptions that they were more likely shared within zone than among zones through animal exchange for breeding.

### 3.4. Sampling Method

Purposive sampling method (judgmental based or researcher based) was applied during breed selection and simple random sampling (lottery method) was used to select an individual animal. The selection of administrative places (zones, districts, and kebeles) was conducted based on previous phenotypic characterization information of the cattle ecotypes. A list of animals that have the mentioned phenotypic characteristics was found from the selected kebeles. This list was used as a sampling frame for the study. Individual animals were selected using simple random sampling from the sampling frame in all the study areas [12, 13].

### 3.5. Sample Collection

Blood samples were collected from 24 unrelated animals of each cattle breed using 4 ml EDTA-coated vacutainer tubes. It is recommendable to study diversity within breed 20–30 range [12, 13] samples. From the three study areas, about 72 blood samples were collected. Then, after gently mixing, collected blood samples were placed in an Ice box and transported to National Agricultural Biotechnology Research Center, Holeta, Ethiopia, and stored at −200°C until DNA extraction.

### 3.6. DNA Extraction

DNA extraction from blood samples was conducted according to the standard salting-out protocol [14]. A 500 *μ*l blood sample was transferred into a 2 ml Eppendorf tube; then 800 *μ*l of lysis buffer was added to each tube (repeated until a white pellet formed).

### 3.7. Determination of DNA Concentration and Quality

The extracted genomic DNA concentration was checked by Nano drop (Nano Drop® ND-8000). DNA quality was checked using gel electrophoresis by loading 5 *μ*l sample DNA on a 1% agarose gel at 100v for one hour. The gel was stained with gel red and visualized under UV light gel documentation system (Figure S2).

### 3.8. Polymerase Chain Reaction (PCR)

A total of 16 bovine-specific microsatellite markers were used for cattle genetic characterization (Table 1) [15]. Polymerase chain reaction (PCR) was performed by touch down method with two steps. The 1st step was initial denaturation at 950C for 3 minutes. Then, it was followed by 20 cycles of denaturation of 950C for 20 sec, annealing begins at 790C and ends at 52.40C for 45 sec, and extension at 720C for 1 minute. The annealing temperature was decreased by 10C until it reached 52.40 C. At the second cycle, denaturation of 940C for 20sec, with 10 cycles, 52.40C for 45 sec, and 720C for 1 minute was applied. The final extension 720C for 10 minutes was applied in all reactions. The final volume of the reactions was 10 *μ*l. The polymerase chain reaction component was done in a total of 10 *μ*l, which included 5 *μ*l DreamTaq PCR master mix 2X, 10 *μ*M forward primer (0.25 *μ*l), 10 *μ*M reverse primer (0.25 *μ*l), 20 ng templates DNA (0.5 *μ*l), and nuclease-free water (4 *μ*l) and control reactions with no DNA template has been prepared to check for DNA contamination and primer dimers. At the end of the reaction, the PCR products were stored at +40C.

### 3.9. Gel Electrophoresis

**Figure 1:**
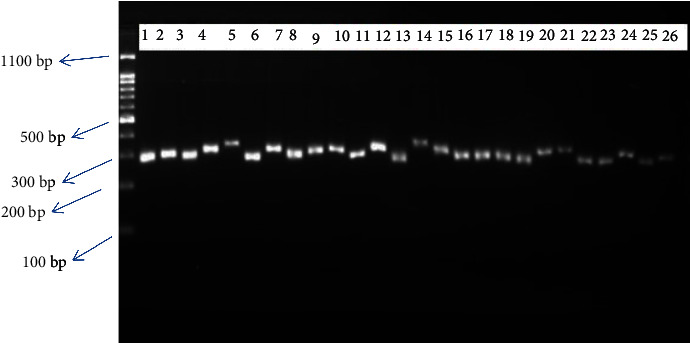
PCR product of selected Ethiopian indigenous cattle breeds using microsatellite markers (ILST006 primer using 100 bp).

**Figure 2:**
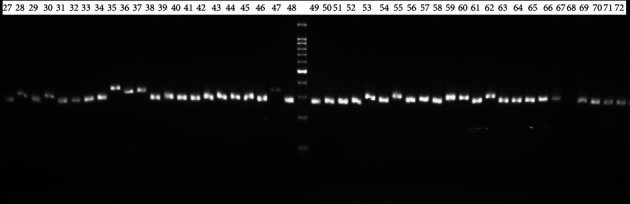
PCR product of selected Ethiopian indigenous cattle breeds using microsatellite markers (ILST006 primer using 100 bp).

To assess amplification, 6 *μ*l of the PCR product was loaded on 2% agarose gel prepared by dissolving 2 g of agarose in 100 ml 1XTAE buffer, staining with gel red. Electrophoresis was carried out at 80 V for 3 : 00 hrs. After completion of electrophoresis, the gel pictures were taken under UV Tran's illuminator by Biodoc analysis with a digital cannon camera (Figures 1 and 2).

### 3.10. Data Scoring and Statistical Analysis

The clear and visible amplified bands of 72 genotype of selected Ethiopian indigenous cattle using microsatellite markers were scored using the PyElph 1.4 software [16].

Genetic variability was measured by estimating observed (Ho) and expected (He) heterozygosities [17]. The Polymorphic Information Content (PIC), the unbiased *F*-statistics [18], and the Analysis of Molecular Variance (AMOVA) were determined using the GenAlex software version 6.5 [19] and Power Marker. Pairwise FST (proportion of genetic variability due to population substructuring) values among pairs of populations were computed for all populations using GenAlex software version 6.5. Using the POPGEN version 1.31 software package [20] and the observed heterozygosity was done according to the algorithm of Levene [21].

Arlequin 3.5 was used to estimate basic frequency-based population genetic parameters such as gene diversity, the total number of alleles (No), Ne, breed private alleles (PAs), allele sizes, and allele ranges (in base pairs) [22]. Principal Coordinate Analysis (PCoA) was used to infer genetic similarities using the covariance matrix of Nei's genetic distance (DA) and unbiased genetic distances (DS) measurements. Dendrograms were created from pair-wise matrices of DA using the agglomerative hierarchical clustering unweighted pair group with arithmetic mean (UPGMA) method and DARwin vars to visualize evolutionary relationships among breeds. The Dendro UPGMA online application [23] was used to design trees, which were then displayed in Tree View [24]. Bootstraps of 1000 replicates were used to establish confidence statements about the breed groups and to test the clusters' dependability.

HP-Rare 1.1software were used to calculate the rarified allelic richness (Ar) and private rarified allelic richness (Arp) [25].

**Figure 3:**
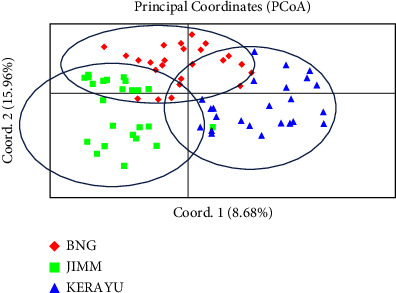
Principal coordinates analysis (PCOA) of 72 genotypes using 16 microsatellite markers.

With independent allele frequencies and an admixture model (burn of 50000, followed by 100000 MCMC iterations with 10 replicate runs for each), the population structure analysis was carried out (1–10 K). Its most appropriate *K* value was identified based on the computed *K* value, and *k* = 3 was discovered to be the most likely number of clusters to partition the 72 genotypes into three (Figure 3) using the STRUCTURE harvester program [26]. The CLUMPAK tool, developed by Kopelman et al. [27], was used to determine the best alignment from the STRUCTURE data, and the CLUMPAK result revealed genetic mixing and no clear geographic origin-based population structuring.

## 4. Results

### 4.1. Microsatellite Locus Polymorphism

The 16 loci yielded a total of 131 alleles, with an average of 8.18 alleles per locus. The No ranged from 5 (RM067) to 12 (ETH3 and TGLA122) and MAF ranged from 0.23 (ETH10) to 0.61 (TGLA126) with a mean of 0.39 per locus. Observed heterozygosity (Ho) varied between 0.00 (ETH10, TGLA126, ILST006, CSSM66, BM2113, SPS115, RM067, TGLA227, TGLA53, and BM1818) and 0.297 (ETH3), while expected heterozygosity (He) ranged from 0.5 (RM67) to 0.75 (ETH10). Ho (0.023) was lower than He (0.66) and this showed that the populations are carrying out inbreeding (Table 1).

The average allelic richness (AR) was 4.57, ranging from 3.33 (TGLA126) to 6 (ETH3), and private alleles (PA) also ranged from 0.66 (BM1818) to 3.66 (INRA023) with an average of 2.1 per locus, respectively, out of 131 alleles, 33 (25%) of PA was unique to specific breeds. The gene diversity (GD) ranged from 0.55 (TGLA126) to 0.86 (ETH10), and the average gene diversity over all loci was 0.74. The 16 microsatellites showed a highly significant (*P* < 0.001) deviation from the Hardy‒Weinberg equilibrium (Table 2).

### 4.2. Genetic Diversity of Population

Comparatively the mean number of alleles observed in this study ranged from Na = 4.94 (Bonga) to Na = 5.56 (Kerayu), and the medium mean number of alleles observed was in Jimma cattle populations. Secondly, Kerayu populations (Ne = 3.4) score the highest effective number of alleles than Jimma (Ne = 3.2), Bonga (Ne = 3.06) and with a total mean of Ne = 3.23. In Kerayu cattle populations, the highest number of private alleles were also observed (PA = 4.13), Jimma (PA = 1.13), and Bonga (PA = 1.0) with a total mean of PA = 2.1. Shannon's information index ranged from I = 1.26 (Bonga and Jimma) to I = 1.34 (Kerayu) with an average mean of I = 1.29. The other things listed above the table were Ho and He. The Ho ranged from 0.044 (Kerayu), 0.016 (Jimma), and 0.011 (Bonga), respectively (Table 3).

Allelic richness ranged from AR = 3.88 (Bonga) to (AR = 5.56) Kerayu. Expected heterozygosity (He) had the highest mean value of 0.658 in (Bonga) and the lowest in Jimma (0.63). Observed heterozygosity (Ho) had a mean value of 0.011 (Bonga) to 0.044 (Kerayu). In Kerayu cattle populations, the highest number of private alleles were also observed (PA = 4.13), Jimma (PA = 1.13), and Bonga (PA = 1.0). Shannon's information index ranged from I = 1.26 (Bonga and Jimma) to I = 1.34 (Kerayu) with an average mean of I = 1.29. The Ho ranged from 0.044 (Kerayu), 0.016 (Jimma), and 0.011 (Bonga), respectively. The average mean number of alleles was from Na = 4.94 (Bonga) to Na = 5.56 (Kerayu), and allelic richness ranged from AR = 3.88 (Bonga) to (AR = 5.56) Kerayu. Expected heterozygosity (He) had the highest mean value of 0.658 in (Bonga) and the lowest in Jimma (0.63). Observed heterozygosity (Ho) had a mean value of 0.011 (Bonga) to 0.044 (Kerayu) (Table 3).

### 4.3. Analysis of Molecular Variance and Gene Flow

Population variance could be classified based on AMOVA among individuals within populations variability (84%) and (13%) variation among populations/among individuals and 3% within the individual. The analysis also confirmed the presence of considerable gene flow (1.69) among subpopulations (Table 4).

### 4.4. Genetic Distance and Genetic Differentiation between Populations

The genetic differentiation between populations ranged from 0.100 between (Bonga and Jimma) to 0.120 (Kerayu and Bonga). The highest GD (0.120) was between Kerayu and Bonga populations, and the lowest genetic differentiation was observed between Jimma and Bonga populations (Table 5). This might be due to geographical locations and types of population/ecotype used. The highest (0.46) genetic distance was observed between Kerayu and Bonga cattle breeds. However, the lowest (0.407) genetic distance was determined between Jimma and Bonga cattle breeds.

### 4.5. Cluster Analysis of Genotype in Three Ethiopian Indigenous Cattle

The unweighted neighbor-joining cluster analysis (UPGMA) categorized the 72 genotypes into three major clusters (Cl-I, Cl-II, and Cl-III). From Figure 2, three types of clustering, dark-red, blue, and green color clusters showed Bonga cattle, Jimma, and Kerayu cattle ecotypes, respectively. Cl-I (36 percent), Cl-II (48.2 percent), and Cl-III (16 percent) of the overall population make up the three clusters, respectively. The first cluster, which contained 24 genotypes from all populations excluding Bonga and Jimma cattle genotypes, was the major cluster, whereas the second cluster contained 24 genotypes except the Bonga genotype. The third cluster contains 12 genotypes. The first cluster consists of 33% genotype from Kerayu, 1.4% genotype from Bonga, and 1.4% genotype from Jimma cattle, respectively.

**Figure 4:**
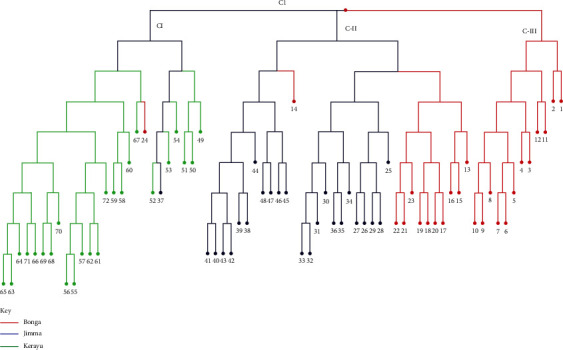
Hierarchical clustering of three Ethiopian indigenous cattle using NJ dendrogram.

Cluster-II consists of 33% and 15.2% from Jimma and Bonga, respectively. Cluster three consists of only genotype from Bonga (16%). Genotypes from Bonga are mainly found in all three clusters, especially found in cluster-II (Jimma) (Figure 4).

**Figure 5:**
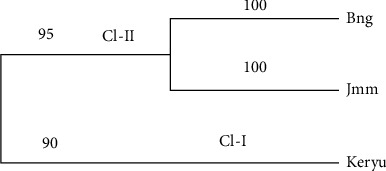
Dendrogram showing the genetic diversity and dissimilarity among the three selected Ethiopian indigenous cattle generated by 16 microsatellite markers (with observations Bng = Bonga, Jmm = Jimma, Keryu = Kerayu).

Figure 5 indicates that there is high gene flow between the two breeds (Bonga and Jimma). Population grouping was also carried out based on the UPGMA method to determine the relationship among the three selected indigenous cattle groups. According to the analysis, the populations are divided into two major clusters. Kerayu (I) is categorized under cluster one and both Jimma and Bonga (II) are categorized under the second cluster. Clustering patterns indicated that populations from geographically adjoining regions like Bonga and Jimma are subclustered together (Figure 5).

### 4.6. Principal Coordinate Analysis (PCOA) and Population Structure

Principal coordinate analysis was also used to look at the genetic relatedness of 72 genotypes (PCOA). The pattern of genotype distributions on a two-dimensional plot showed separate clustering of populations based on the geographic locations and revealed a high pattern of grouping. The PCoA analysis displayed in Figure 3 below confirms and was complementary to the result of the NJ cluster analysis shown in Figure 4.

**Figure 6:**
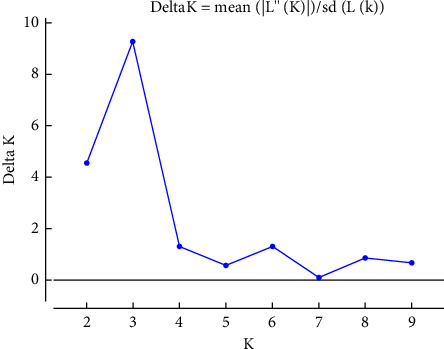
Population structure of three cattle breeds obtained by STRUCTURE analysis (*K* = 3). Based on Evano et al. [28].

**Figure 7:**
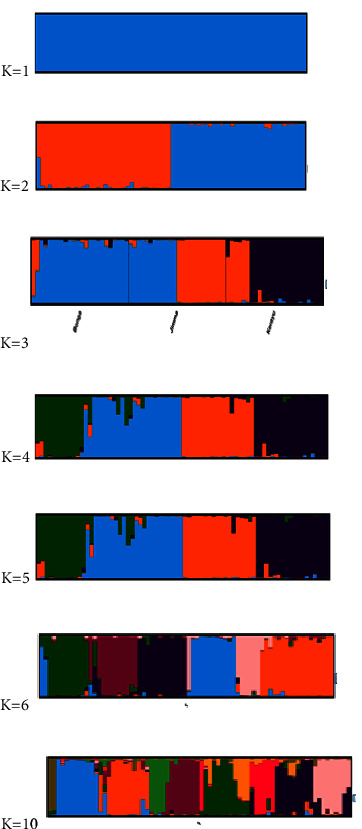
Population structure of three cattle breeds obtained by STRUCTURE analysis (*K* = 1, 2, 3,4,5,6, and 10), where each color represents a different cluster.

With independent allele frequencies and an admixture model (burn of 50000, followed by 100000 MCMC iterations with 10 replicate runs for each), the population structure analysis was carried out (1–10 K). In the method (K = m|L″(*K*)|/s[L(*K*)] published by Evano et al. [28], the suitable number of clusters was discovered using *K* values that reflected the proportion of change in the logarithmic probability Pr(X|K) of data between *K* values (28) (Figure 6). Its most appropriate K value was identified based on the computed K value and *k* = 3 were discovered to be the most likely number of clusters to partition the 72 genotypes into three (Figure 7).

## 5. Discussion

### 5.1. Genetic Diversity

According to the classification of Botstein et al. [29], the highly informative markers have PIC values >0.50, the reasonably informative markers have PIC values between 0.25 and 0.50, and the slightly informative markers have PIC values <0.25. In this study, all sixteen microsatellite loci used to profile genetic diversity of 72 genotype were found to be highly polymorphic with PIC >0.5. For evaluating genetic differences between animal breeds, all 16 microsatellite markers exceeded the FAO's suggested minimum threshold of five alleles per locus [13, 15]. Öner et al. [30] reported average mean PIC value was 0.87. The average means PIC value (0.70) of the present study indicated the markers used in this study were highly informative and the availability of high allelic variation in the marker loci and their distribution within the population's genome. The result of the present study was also used for genetic diversity analysis. In general, the PIC of this study (0.70) was higher than that of the previous one reported by Gororo et al. [15] (0.664). Demir and Balcioglu [31] and also Hussain et al. [32] reported a PIC value of 0.82 using microsatellite markers, which was greater than the PIC value of this study. The variation in PIC value could be due to a higher number of samples/breed types and an increased number of markers and implying the high discriminating ability of the markers.

Gororo et al. [15] found 119 alleles in total, with an average of 7.4 alleles per locus and 34 private alleles. Hussain et al. [32] also reported a total of 476 alleles with a 22.33 average mean of alleles per locus using 21 microsatellite markers. Jakaria et al. [32] reported 46 alleles in four microsatellite markers with an average mean of 11.5 per locus. Other studies by Özşensoy et al. [33] reported 269 alleles in 20 markers with a 13.45 average mean of alleles per locus. Öner et al. [30] reported a total of 545 alleles in 22 microsatellite markers with an average mean of 23.14 alleles per locus, and this observed number of alleles difference might be due to the number of genotypes, number of the marker, and breed number used.

### 5.2. Genetic Diversity Analysis along with Populations

Previously, both Dadi et al. [8] and Gororo et al. [15] reported similar observed and expected heterozygosity across the populations. Agung et al. [34] also detected 0.66, 0.68, Ho and He, respectively. But, in this study, the level of observed and expected heterozygosity across the study populations was different, the Ho and He of this study were lower than the study reported by previous workers [8, 35].

Demir and Balcioğlu [31] reported that the Ho and He mean values were 0.63 and 0.74, respectively, due to the increased number of microsatellite markers and individual samples. But, in this case, Ho and He values were 0.023 and 0.651, respectively.

The number of individuals sampled per population and the number of populations studied might be the main cause for the higher numbers of Ho and He in Dadi's et al. [8] study and others. This difference might be due to factors like Null alleles, assortative mating, the Wahlund effect, selection against heterozygotes, inbreeding, or a combination of all of these reasons that can all explain this state [15, 35].

In this study, the mean number of alleles for each breed (Bonga, Jimma, and Kerayu) was 4.94, 5.063, and 5.56, respectively. Demir and Balcioğlu [31] reported that the mean number of alleles for four breed types such as Turkish Grey steppe (7.95), Eastern Anatolian Red (7.15), Anatolian Black (8.45), and HF (7.1). Agung et al. [34] also reported the mean number of alleles for 11 different cattle breeds was 6.28. The variation of mean alleles in this study and the previous study might be due to the increased number of microsatellite markers and the number of breed types used for the study. Gororo et al. [15] reported a mean number of alleles (Na) of 5.16, which was similar to this study, microsatellite markers based on characterization of selected Ethiopian cattle like Bonga, Jimma, and Kerayu and Na detected were 5.18.

The other measure of gene diversity is the Shannon information index (I), if Shannon's information index value is close to one or above, it indicates that there is variation in the tested populations and that the markers are suitable for studying diversity [36]. The value obtained in this study ranged from I = 0.86 to I = 1.63 with an average mean of 1.28. This implies that selected Ethiopian indigenous cattle have genetic diversity.

According to Zerabruk et al. [35], allelic richness reported in north Ethiopia of cattle ranged from 5.67 to 6.27 with a mean of 6.23 AR per breed. But, in this study, AR ranged from 3.88 to 5.56 with a mean of 4.5 AR per breed. This showed that there is genetic diversity among populations.

Agung et al. [34] reported a 3.81 effective number of alleles across 11 different breeds using 12 microsatellite markers; however, in this study, 3.23 effective numbers of alleles were detected using 16 microsatellite markers from 3 different Ethiopian indigenous cattle breeds. Hussain et al. [32] reported 6.7 effective numbers of alleles. This showed that the types of breeds and the number of breeds used might be the cause for the variation in an effective number of alleles.

To quantify the genetic variability, in Ethiopian indigenous cattle breeds and 16 microsatellite markers were used resulting in a Fis value (0.966), showing that the selected Ethiopian indigenous cattle have undergone inbreeding. Jakaria et al. [37] reported a Fis value of 0.07 using five microsatellite markers, and it was lower than the Fis value reported in this study. FST values could indicate small (0–0.05), medium (0.05–0.15), high (0.15–0.25), and very high (FST >0.25) genetic differentiation between breeds [38].

The estimated FST value for 3 different cattle breeds was higher than Zimbabwean cattle breeds (0.084) [15]; this finding was comparable with those reported by Sharma et al. [39], 13.3% of FST value in Indian cattle using STR markers and high FST value of this study indicated that 16 microsatellite markers used for 3 breeds/ecotypes were significantly high and are useful indicators of markers that could be powerful tools for genetic differentiation of different breeds. But, lower than Pakistan cattle breeds (0.1456) Rahal et al. [40], and Indonesian cattle breeds (0.243) [39]. Jakaria et al. [37] also revealed a higher number of FST values (0.246) than this study. El-Sayed et al. [41, 42] detected a higher FST (0.236) value from two Egyptian cattle breeds. This FST variation might be due to gene flow and exchange of breeding animals. The FST is higher when the populations are isolated between them.

### 5.3. Analysis of Molecular Variance

In addition, AMOVA shown in genetic variation among breeds was 13%, and 84% within the population, 3% within an individual was observed. Significant genetic differentiation was observed among all 3 different Ethiopian indigenous cattle (Bonga, Jimma, and Kerayu) estimated by FST = 0.129. Dadi et al. [8] reported 1.3% genetic variation among populations and 98.7% genetic variation within populations. Jakaria et al. [37] also reported genetic variation within a population (70.6%) was higher than among populations (29.4%). The genetic variation within the population was higher than among populations this implies that there might be due to interpopulation gene flow, sexual recombination, and mutations.

### 5.4. Cluster Analysis, PCOA, and Population Structure

In clustering, a dendrogram of cluster analysis based on NJ algorism using UPGMA categorized the three ecotypes into three clusters based on the geographical locations (I, II, and III) with different subgroups. Dadi et al. [8] obtained two main clusters using 30 microsatellite markers with 10 indigenous, one HF, and different subgroups formed under two main subgroups. Cervini et al. [43] showed two different clusters of dendrogram-based UPGMA/NJ using 12 microsatellite markers in 11 different cattle breeds. A study by Edea et al. [6] also revealed two main clusters in six Ethiopian indigenous and one Korean cattle breed using SNP markers.

The difference might be due to the number of microsatellites/type of microsatellite used, some ecotypes/breeds, and sample numbers. The clustering model showed that there was a relationship between the patterns of genetic diversity and the geographical origins of the collection. Populations collected from Bonga and Jimma had a strong relationship. Bonga and Jimma cattle were close to each other and the existence of gene flow among the neighboring populations seems possible.

Moreover, this result is also clearly reflected in population structure showing weak admixture of a gene across populations. It revealed the existence of substructuring (*K* = 3) in three populations of Ethiopia. Previously, Jakaria et al. [37] also reported population structure with *k* = 3 in four cattle populations and there were genetic admixtures. This could be likely due to the presence of gene flow between the ecotypes because of the movement of cattle and uncontrolled mating/exchange of breeding animals, migration from one region to another. Grouping of PCoA corresponds with the clustering dendrogram based, which showed conformity result obtained from UPGMA analysis Agung et al. [44].

## 6. Conclusion

In this study, the genetic diversity and population structure of the selected Ethiopian indigenous cattle were covered using highly polymorphic microsatellite markers. The study confirmed the presence of genetic diversity in selected Ethiopian indigenous cattle breeds, indicating the possibility of improvement through breeding and the importance of maintaining diversity by applying appropriate conservation strategies. This study also indicated genetic variation (84%) accounted for within populations, likely due to gene flow, sexual recombination, and mutation. The populations showed moderate genetic differentiation, due to high gene flow. The highest genetic diversity indices were recorded for Kerayu cattle populations, suggesting that this area is a hotspot for genetic diversity studies, sources of important alleles for breeding purposes, and conservation strategies must be applied.

In general, cluster analysis, PCoA, and population structure analysis exhibited moderate grouping of samples. Studying the genetic basis of locally adapted indigenous cattle populations is critical for developing appropriate breeding strategies and programs aimed at improving and conserving their genetic diversity. From this study, the generated information is valuable for the national animal breeding program and conservation purposes.

## Figures and Tables

**Table 1:** Microsatellite markers, their sequence, and chromosomes number location and annealing AT°C using touchdown PCR.

S/n	Loci	Chro	Forward	Reverse	Repeats	AT°C	Size
1	BM1818	23	AGCTGGGAATATAACCAAAGG	AGTGCTTTCAAGGTCCATGC	(GT)13	67.9–52.9	248–276
2	BM1824	1	GAG CAAGGT GTT TTTCCAATC	CATTCTCCAACTGCTTCCTTG	(TG)26	68.4–53.4	170–218
3	BM2113	2	GCTGCCTTCTAC CAA ATA CCC	CTTCCTGAGAGAAGCAACACC	(CA/GT)20	71.3–56.3	116–146
4	CSRM60	10	AAGATGTGATCCAAGAGAGAGGCA	AGGACCAGATCGTGAAAGGCATAG	(CA)17	74.4–59.4	79–115
5	CSSM66	14	AATTTAATGCACTGAGGAGCTTGG	ACACAAATCCTTTCTGCCAGCTGA	(AC)17	74.4–59.4	171–209
6	ETH10	5	GTTCAGGACTGGCCCTGCTAACA	CCTCCAGCCCACTTTCTCTTC TC	(CA)12	76.4–61.4	198–234
7	ETH225	9	GATCACCTTGCCACTATTTCCT	GTGTCTTACATGACAGCCAGCTGCTAC T	(CG)n(TG)4(CA)18	75.2–60.2	132–166
8	ETH3	19	GAACCTGCCTCTCCTGCATTGG	GTGTCTTACTCTGCCTGTGGCCAAGA GG	(GT)26	79–64	90–135
9	ILST006	7	TGTCTGTATTTC TGCTGTGG	ACACGGAAGCGA TCTAAACG	GT)23	67.4–52.4	277–309
10	NRA023	3	GAGTAGAGCTACAAGATAAACTTC	TAACTACAGGGTGTTAGATGAACTC	(AC)	71.3–56.3	194–236
11	RM067	4	TGAGTAATGCAATAGATACAGTATT	GCTTTGGCCATATGAAGAGCTTT	(AC)17	69.3-54-3	83–101
12	SPS115	15	AAAGTGACACAACAGCTTCTCCAG	GTGTCTTAACGAGTGTCCTAGTTTGGCTGTG	(CA)27(TA)n	77.9–62.9	240–270
13	TGLA12	21	AATCACATGGCAAATAAGTACATAC	CCCTCCTCCAGGTAAATCAGC	(AC)n(AT)n	71.3–56.3	133–103
14	TGLA16	20	CTAATTTAGAATGAGAGAGGCTTCT	TTGGTCTCTATTCTCTGAATATTCC	(TG)	65.9–55.9	104–133
15	TGLA27	18	GGAATTCCAAATCTGTTAATTTGCT	ACAGACAGAAACTCAATGAAGCA	(TG)n	69.7–54.7	63–115
16	TGLA53	16	GCTTTCAGAAATAGTTTGCATTCA	TGTCTTATCTTCACATGATATTACAGCA GA	(TG)6(CG)4(TA)	71.8–56.8	147–197

**Table 2:** Genetic diversity parameters for 16 microsatellite loci analyzed in three Ethiopian indigenous cattle breeds.

Loci	Allele in bp	NO	Na	MAF	Ne	AR	PA	PIC	Ho	Fst	P(Fst)	Nm	I	He	Ht	UHe	*F*	*p*	*P*(HWE)
ETH10	194–251	11	7.00	0.23	4.39	5.33	2.33	0.84	0.00	0.12	0.001	1.73	1.63	0.75	0.85	0.76	0.98	0.00	^*∗∗∗*^
TGLA122	141–218	12	6.66	0.30	3.94	6.33	2.33	0.80	0.028	0.09	0.001	2.29	1.56	0.74	0.82	0.75	0.96	0.00	^*∗∗∗*^
INRA023	180–271	10	4.66	0.4	2.87	4.66	3.66	0.74	0.014	0.24	0.001	0.75	1.12	0.57	0.76	0.58	1.0	0.00	^*∗∗∗*^
TGLA126	156–206	6	3.33	0.61	2.10	3.33	1.33	0.50	0.014	0.07	0.034	3.27	0.86	0.51	0.55	0.52	0.97	0.00	^*∗∗∗*^
BM1824	170–230	6	3.66	0.44	2.68	3.66	2.33	0.71	0.00	0.16	0.001	1.23	1.09	0.61	0.73	0.62	1.0	0.00	^*∗∗∗*^
ILST006	284–320	7	4.33	0.47	2.37	3.66	1.33	0.62	0.00	0.21	0.001	0.92	1.02	0.53	0.67	0.54	1.0	0.00	^*∗∗∗*^
CSSM66	175–240	7	4.00	0.44	2.79	4.33	3	0.70	0.00	0.15	0.001	1.41	1.10	0.62	0.73	0.63	1.0	0.00	^*∗∗∗*^
ETH225	140–275	9	6.66	0.31	4.27	4.66	2.33	0.78	0.014	0.09	0.002	2.27	1.55	0.72	0.80	0.73	1.0	0.00	^*∗∗∗*^
BM2113	123–148	7	5.00	0.25	3.43	4	1.33	0.79	0.00	0.13	0.001	1.57	1.37	0.70	0.81	0.72	1.0	0.00	^*∗∗∗*^
SPS115	260–300	7	4.00	0.38	2.77	4.66	1.33	0.73	0.00	0.20	0.001	0.98	1.12	0.60	0.76	0.62	0.96	0.00	^*∗∗∗*^
ETH3	105–155	12	7.00	0.28	4.70	6	3	0.78	0.278	0.04	0.029	4.80	1.64	0.76	0.80	0.78	0.64	0.022	^*∗*^
RM067	85–125	5	3.66	0.51	2.21	3.66	1.66	0.59	0.00	0.21	0.001	0.92	0.92	0.50	0.64	0.51	1.0	0.00	^*∗∗∗*^
TGLA227	64–120	8	6.33	0.42	3.33	5	2.66	0.72	0.00	0.06	0.022	3.66	1.47	0.70	0.74	0.71	1.0	0.00	^*∗∗∗*^
TGLA53	154–200	7	5.00	0.39	3.09	4	1.66	0.64	0.00	0.04	0.135	5.11	1.25	0.66	0.69	0.67	1.0	0.00	^*∗∗∗*^
BM1818	250–278	6	4.33	0.50	2.91	3.66	0.66	0.64	0.00	0.043	0.161	5.61	1.23	0.66	0.68	0.65	1.0	0.00	^*∗∗∗*^
CSRM60	115–140	11	7.33	0.40	3.82	6.33	2	0.72	0.00	0.021	0.689	11.61	1.59	0.73	0.75	0.75	1.0	0.00	^*∗∗∗*^
Mean		**8.18**	**5.18**	**0.39**	**3.23**	**4.57**	**2.1**	**0.70**	**0.023**	0.129	0.001	3.01	1.28	**0.66**	**0.73**	**0.65**	0.96	0.00	^*∗∗∗*^

Allele size range (in bp), allelic richness (AR), private alleles (PA) (No, observed number of alleles; MAF, major allele frequency, Na, mean number of alleles; Ne, the effective number of alleles), marker in informativeness (PIC, polymorphism information content), heterozygosity (Ho, observed; He, expected; Ht, total). ^*∗*^HWE: ns stands for “not significant,” ^*∗∗*^*P* < 0.01, ^*∗∗∗*^*P* < 0.001, ^*∗*^*P* < 0.05, ^*∗∗*^*P* < 0.01, ^*∗∗∗*^*P* < 0.001.

**Table 3:** Sixteen microsatellite markers used to estimate genetic diversity parameters in three Ethiopian cattle breeds.

Breeds	N	Na	Ne	PA	I	Ho	He	AR	%*P*
Bonga	24	4.94	3.06	1	1.26	0.011	0.658	3.88	100
Jimma	24	5.063	3.2	1.3	1.26	0.016	0.635	4.06	100
Kerayu	24	5.56	3.4	4.13	1.34	0.044	0.658	5.56	100
Mean		5.18	3.23	2.1	1.29	0.023	0.651	4.5	100

NA; mean number of alleles, and AR (allelic richness). NA; mean number of alleles, and AR (allelic richness). Na: stands for the average number of alleles. PA stands for private alleles and Ne stands for an effective number of alleles. The Shannon information index or I; He stands for expected heterozygosity, while % *P* stands for polymorphic loci proportion.

**Table 4:** Summary of AMOVA.

Source	Df	SS	MS	Est. var	Variation (%)	*F* statistics	*P* value
Among popns	2	100.042	50.021	0.816	13	FST = 0.129	0.001
Among individual	69	750.313	10.874	5.343	84	Fis = 0.966	0.001
Within individual	72	13.500	0.188	0.188	3	FIT = 0.97	0.001
Total	143	863.854		6.346	100		
Nm						**1.69**	

Df = Degree of freedom, SS, the sum of a square, Ms, means of a square, NM = gene flow.

**Table 5:** Pairwise population differentiation using Nei genetic distance (above diagonal) and FST (below diagonal) values.

Breed	Bonga	Jimma	Kerayu
Bonga	0.00	0.400	**0.460**
Jimma	**0.100**	0.00	**0.450**
Kerayu	0.120	0.110	0.00

## Data Availability

The corresponding author will provide the datasets produced or analyzed during the current investigation upon reasonable request.

## Abbreviations

AR: Allelic richness
PA: Private alleles
Masl: Mean above sea level
PCOA: Principal coordinate analysis
UPGM: Unweighted pairwise group method
I: Shannon information index
PIC: Polymorphic information content
HWE: Hardy‒Weinberg equilibrium
AMOVA: Analysis of molecular variance
NJ: Neighbor joining tree
MAF: Major allele frequency
GDP: Gross domestic product.

## Supplementary Materials

Supplementary Materials Figure S1: Study Area. Figure S2: Image of genomic DNA. Table S1: List of sample collection site used for the study and their geographical origins.
