# Supplementary material for: Genetic Diversity and Population Structure of Selected Ethiopian Indigenous Cattle Breeds Using Microsatellite Markers
Source: Genet Res (Camb). 2023 Jan 14;2023:1106755. doi: 10.1155/2023/1106755 (PMC9867593; doi:10.1155/2023/1106755)
Supplement: Supplementary Materials — Figure S1: Study Area. Figure S2: Image of genomic DNA. Table S1: List of sample collection site used for the study and their geographical origins. [file 1106755.f1.docx]

Supplementary Information


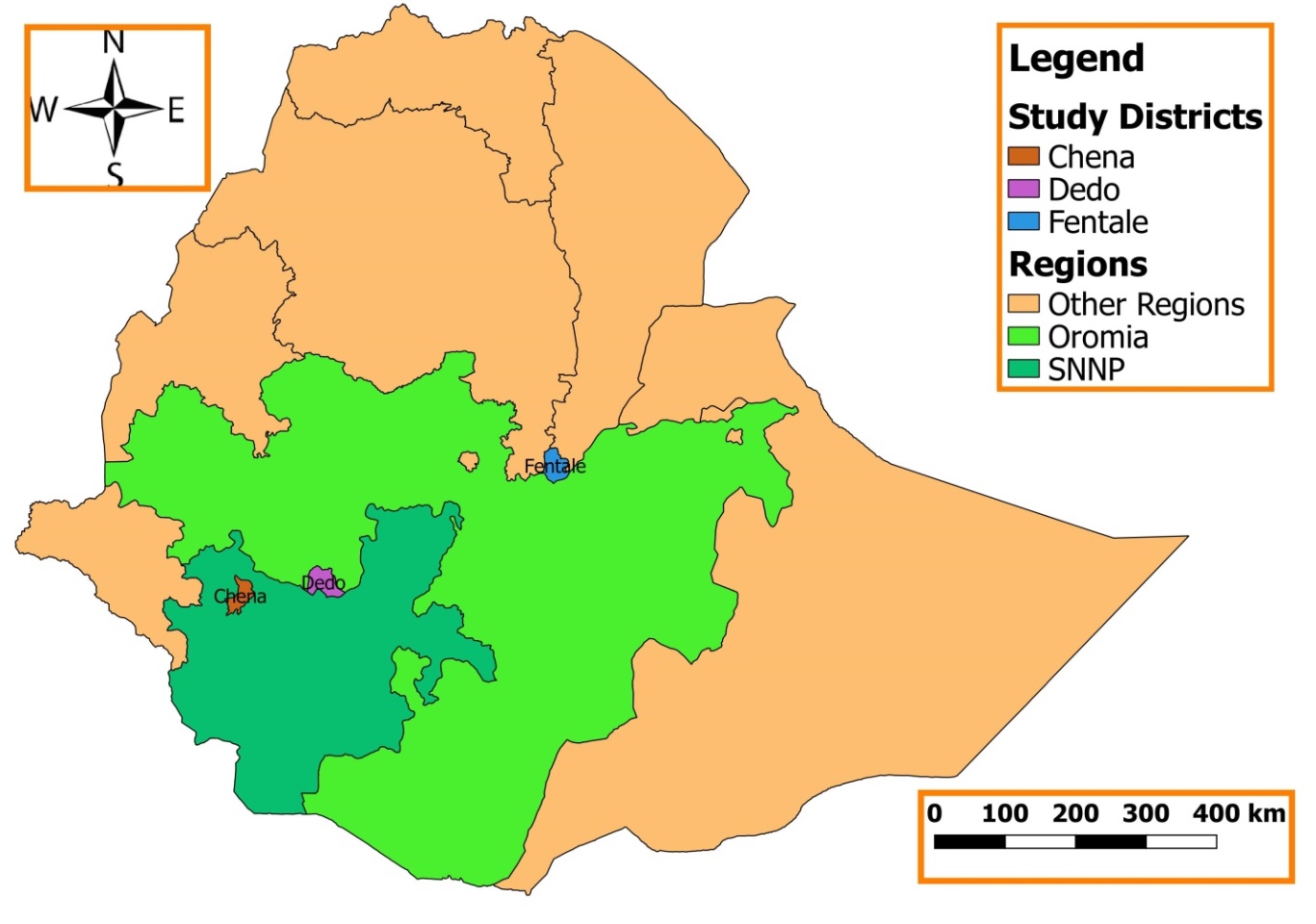


**Figure S 1**: Study Area


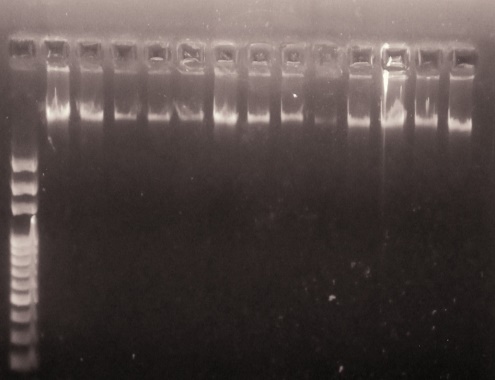


**Figure S 2**: Image of genomic DNA

**Table S 1:** List of sample collection site used for the study and their geographical origins

| S/n | Code | Zone | Woreda | Kebele | Latitude | Longitude | Altitude(M.asl) |
| --- | --- | --- | --- | --- | --- | --- | --- |
| 1 | 01 | Bonga | Chena | Wota | 7.2-35-64N | 35-75-15-43E | 1851-1900 masl |
| 2 | 02 | Bonga | Chena | Wota | 7.2-35-64N | 35-75-15-43E | “ |
| 3 | 03 | “ | “ | “ | “ | “ | “ |
| 4 | 04 | “ | “ | “ | “ | “ | “ |
| 5 | 05 | ‘’ | “ | “ | “ | “ | “ |
| 6 | 06 | ‘’ | “ | “ | “ | “ | “ |
| 7 | 07 | “ | “ | “ | “ | “ | “ |
| 8 | 08 | “ | “ | “ | “ | “ | “ |
| 9 | 09 | “ | “ | “ | “ | “ | “ |
| 10 | 010 | “ | “ | “ | “ | “ | “ |
| 11 | 011 | “ | “ | “ | “ | “ | “ |
| 12 | 012 | “ | “ | “ | “ | “ | “ |
| 13 | 013 | “ | “ | Warkalem | “ | “ | 1765 |
| 14 | 014 | “ | “ | Warkalem | “ | “ | “ |
| 15 | 015 | “ | “ | “ | “ | “ | “ |
| 16 | 016 | “ | “ | “ | “ | “ | “ |
| 17 | 017 | “ | “ | “ | “ | “ | “ |
| 18 | 018 | “ | “ | “ | “ | “ | “ |
| 19 | 019 | “ | “ | “ | “ | “ | “ |
| 20 | 020 | “ | “ | “ | “ | “ | “ |
| 21 | 021 | “ | “ | “ | “ | “ | “ |
| 22 | 022 | “ | “ | “ | “ | “ | “ |
| 23 | 023 | “ | “ | “ | “ | “ | “ |
| 24 | 024 | “ | “ | “ | “ | “ | “ |
| 25 | 025 | Jimma | Dedo | Tito | 7.5-06-379-N | 36-88-46-11E | 3360 |
| 26 | 026 | Jimma | Dedo | Tito | 7.5-06-379-N | 36-88-46-11E | 3360 |
| 27 | 027 | “ | “ | “ | “ | “ | “ |
| 28 | 028 | “ | “ | “ | “ | “ | “ |
| 29 | 029 | “ | “ | “ | “ | “ | “ |
| 30 | 030 | “ | “ | “ | “ | “ | “ |
| 31 | 031 | “ | “ | “ | “ | “ | “ |
| 32 | 032 | “ | “ | “ | “ | “ | “ |
| 33 | 033 | “ | “ | “ | “ | “ | “ |
| 34 | 034 | “ | “ | “ | “ | “ | “ |
| 35 | 035 | “ | “ | “ | “ | “ | “ |
| 36 | 036 | “ | “ | “ | “ | “ | “ |
| 37 | 037 | “ | “ | Keta | “ | “ | “ |
| 38 | 038 | “ | “ | Keta | “ | “ | 2890 |
| 39 | 039 | “ | “ | “ | “ | “ | 2890 |
| 40 | 040 | “ | “ | “ | “ | “ | “ |
| 41 | 041 | “ | “ | “ | “ | “ | “ |
| 42 | 042 | “ | “ | “ | “ | “ | “ |
| 43 | 043 | “ | “ | “ | “ |  | “ |
| 44 | 044 | “ | “ | “ | “ | “ | “ |
| 45 | 045 | “ | “ | “ | “ | “ | “ |
| 46 | 046 | “ | “ | “ | “ | “ | “ |
| 47 | 047 | “ | “ | “ | “ | “ | “ |
| 48 | 048 | “ | “ | “ | “ | “ | “ |
| 49 | 049 | East shoa | Fenatalle | Tiyo | 8.75-31-63N | 39-84-85-11E | 800-1100 |
| 50 | 050 | East shoa | Fentalle | Tiyo | 8.75-31-63N | 39-84-85-11E | 800-1100 |
| 51 | 051 | “ | “ | “ | “ | “ | “ |
| 52 | 052 | “ | “ | “ | “ | “ | “ |
| 53 | 053 | “ | “ | “ | “ | “ | “ |
| 54 | 054 | “ | “ | “ | “ | “ | “ |
| 55 | 055 | “ | “ | “ | “ | “ | “ |
| 56 | 056 | “ | “ | “ | “ | “ | “ |
| 57 | 057 | “ | “ | “ | “ | “ | “ |
| 58 | 058 | “ | “ | “ | “ | “ | “ |
| 59 | 059 | “ | “ | “ | “ | “ | “ |
| 60 | 060 | “ | “ | “ | “ | “ | “ |
| 61 | 061 | “ | “ | “ | “ | “ | “ |
| 62 | 062 | “ | “ | “ | “ | “ | “ |
| 63 | 063 | “ | “ | “ | “ | “ | “ |
| 64 | 064 | “ | “ | “ | “ | “ | “ |
| 65 | 065 | “ | “ | “ | “ | “ | “ |
| 66 | 066 | “ | “ | “ | “ | “ | “ |
| 67 | 067 | “ | “ | “ | “ | “ | “ |
| 68 | 068 | “ | “ | “ | “ | “ | “ |
| 69 | 069 | “ | “ | “ | “ | “ | “ |
| 70 | 070 | “ | “ | “ | “ | “ | “ |
| 71 | 071 | “ | “ | “ | “ | “ | “ |
| 72 | 072 | “ | “ | “ | “ | “ | “ |
